# Quantification of retinal pigment epithelial phenotypic variation using laser scanning cytometry

**Published:** 2010-06-16

**Authors:** L.M. Hjelmeland, A. Fujikawa, S.L. Oltjen, Z. Smit-McBride, D. Braunschweig

**Affiliations:** 1 Department of Ophthalmology, School of Medicine, University of California at Davis, Davis, CA; 2 Department of Internal Medicine, Division of Rheumatology, School of Medicine, University of California at Davis, Davis, CA; 3 Department of Ophthalmology, Nagasaki University, Nagasaki, Japan

## Abstract

**Purpose:**

Quantifying phenotypic variation at the level of protein expression (variegation) within populations of retinal pigment epithelium (RPE) cells may be important in the study of pathologies associated with this variation. The lack of quantitative methods for examining single cells, however, and the variable presence of pigment and/or lipofuscin complicate this experimental goal. We have applied the technique of laser scanning cytometry (LSC) to paraffin sections of mouse and human eyes to evaluate the utility of LSC for these measurements.

**Methods:**

Mouse eyes were perfusion fixed in 4% paraformaldehyde and embedded in paraffin. Postmortem human eyes were fixed and dissected to obtain a 9-mm punch, which was then embedded in paraffin. A laser scanning cytometer equipped with violet, argon, and helium-neon lasers and the detectors for blue, green, and long red were used to record the fluorescence of each individual cell at all three wavelengths. Raw data were recorded and processed using the WinCyte software. Individual nuclei were identified by the fluorescence of the 4’,6-diamidino-2-phenylindole (DAPI) nuclear counterstain. Next, RPE cells were uniquely identified in the green channel using an anti-retinal pigment epithelium-specific protein 65 kDa (anti-RPE65) monoclonal antibody with an Alexa Fluor 488-labeled secondary antibody. Mn-superoxide dismutase (MnSOD) was quantified in the long-red channel using an anti-MnSOD antibody and an Alexa Fluor 647-labeled secondary antibody. MnSOD^+^ and RPE65^+^ cells exhibited peaks in the plot of fluorescence intensity versus cell number, which could be characterized by the mean fluorescence intensity (MFI), the coefficient of variation (CV), and the percentage of total RPE cells that were also labeled for MnSOD.

**Results:**

RPE cells can be uniquely identified in human and mouse paraffin sections by immunolabeling with anti-RPE65 antibody. A second antigen, such as MnSOD, can then be probed only within this set of RPE. Results are plotted primarily with the population frequency diagram, which can be subdivided into multiple regions. The data collected for each region include the MFI, the CV, and the number of cells that are immunolabeled in that region. Background interference from pigment or autofluorescent material can be successfully overcome by elevating the concentrations of fluorescent secondary antibodies. In the human and mouse eyes, age-related changes in MFI, CV, and percent RPE cells immunolabeled for MnSOD were observed.

**Conclusions:**

The extent of the variability of gene expression in RPE cells at the protein level can be quantified by LSC. Relative changes in the MFI, the CV, and/or percentage of RPE cells double labeled for a second antigen quantify the changes observed. The analysis of these data also suggest whether the effects observed are related to local changes in transcription (alterations of CV) or major changes of protein expression (MFI), which are likely to be due to changes in the chromatin structure. The changes of these variables with age suggest that the observed age-related variegation is primarily due to changes in the chromatin structure in individual cells.

## Introduction

**Figure 1:**
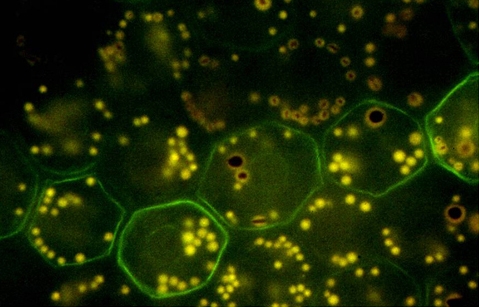
Bovine retinal pigment epithelium (RPE) immunolabeling for vimentin. Whole mount of a bovine retinal pigment epithelium monolayer immunolabeled for the intermediate filament protein vimentin to illustrate a mosaic pattern of protein expression. Vimentin has a circumferential distribution in the peripheral cytoplasm (green) within a row-like subset of retinal pigment epithelium cells. The tissue shown here is from the tapetal region of the cow eye, which has relatively few melanosomes (brown granules), lipofuscin (yellow granules), and combined melanolipofuscin granules [1].

Genetic and phenotypic variation of the retinal pigment epithelium (RPE) within an individual eye is found in humans as well as laboratory animals [1]. The sources of these variations are complex and include both genetic and nongenetic mechanisms. Our laboratory is currently studying epigenetic regulation of gene expression, and we have explored laser scanning cytometry (LSC) as a means of quantifying the phenotypic variation of Mn-superoxide dismutase (MnSOD) protein levels found in individual RPE cells. Several previous publications have documented phenotypic variation in protein content and cellular morphology in the RPE. In a 1996 study [2] bovine retinal pigment epithelial cells in situ exhibited a variation in the expression of vimentin. Figure 1 is an image taken from the review by Burke and Hjelmeland [1].

**Figure 2:**
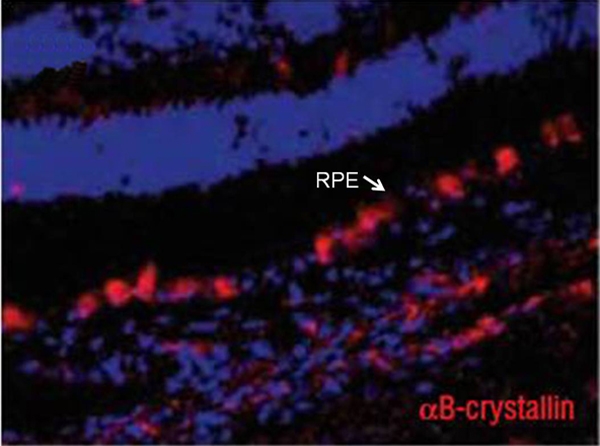
Immunolabeling for αB-crystallin in AMD. αB-crystallin is expressed in retinal pigment epithelium cells in eyes with early and advanced age-related macular degeneration. αB-crystallin immunolabeling is seen in cells of the retinal pigment epithelium (RPE) layer, frequently near drusen in eyes with early dry age-related macular degeneration (AMD). αB-crystallin is more widespread throughout the RPE of eyes with more advanced dry AMD and is frequently seen in RPE cells lying above drusen. The arrow is pointing at the retinal pigment epithelium. Original magnification of the image was 20× [4].

Guidry et al. [3] studied the expression of α smooth muscle actin as well as vimentin in a series of human eyes. A larger study on human eyes with age-related macular degeneration (AMD) and age-matched controls investigated the expression of αB-crystallin as a marker for AMD [4]. αB-crystallin was primarily expressed in the RPE in relation to pathologic features of the tissue and not as a function of age. Figure 2 is from this study and illustrates phenotypic variation in the expression of αB-crystallin in the RPE of an eye from a donor with an early state of dry AMD.

Because autofluorescence of individual cells can also be understood as a phenotype, a specific excitation wavelength combined with an appropriate filter leads to observable differences among RPE cells. With the advent of adaptive optics and confocal laser scanning ophthalmoscopy, it is now possible to directly measure the autofluorescence of individual RPE cells in vivo [5–8].

Does phenotypic variation of the RPE have functional consequences? An early paper by Mullen and LaVail [9] clearly showed that the presence of genes for one type of inherited retinal degeneration in the rat RPE leads to the degeneration of adjacent photoreceptors. These authors used a powerful experimental approach where embryonic cells from two different strains of rats (containing wild type or mutant allele) were combined to form an aggregation chimera at the blastula stage of development. Combining cells from pigmented and unpigmented strains allowed visual inspection of the distribution of genotypes in the RPE. In the adult animal this results in a mosaic pattern of the RPE cell layer and the corresponding mosaic pattern of degeneration in the adjacent photoreceptors. Similar approaches have been used in both the mouse and the zebrafish to show that altered gene expression in the RPE can lead to pathology in the adjacent retina [10–13]. These types of effects on neighboring cells are called noncell-autonomous to indicate that factors arising in the cellular environment (i.e., the RPE) are essential for the observed phenotype (i.e., photoreceptor degeneration). Cell autonomous forms of retinal degeneration proceed directly from the expression of mutant genes within photoreceptors.

What differentiates mosaicism and phenotypic variation? RPE monolayers composed of regions of cells that have different genotypes are defined as mosaics. This terminology is used in developmental biology and more specifically in developmental studies of the RPE in zebrafish [10,11,14], mice [15], and humans [16]. RPE mosaics are not simple patchworks or islands of cells because mixing as well as clonal expansion occurs during development [15]. Many mosaics have a large number of singletons (individual cells with varying genotypes) [17].

Larger patches of cells with a given genotype usually occur more frequently in the mid-periphery and periphery of the RPE monolayer [12,15]. Genetic mechanisms leading to the formation of mosaics fall into several categories that include: 1) experimental chimeras, 2) mutations, deletions, and aneuploidies, and 3) X chromosome inactivation. The experimental aggregation of blastulas from organisms with different genotypes leads to the formation of an aggregation chimera [12]. Combining cells from pigmented and unpigmented strains, for example, allows visual inspection of the distribution of genotypes in the RPE [17]. The mouse and zebrafish are widely studied in this fashion [11,12,14]. The use of aggregate chimeras to observe migration and clonal expansion during mouse RPE development has been reviewed [12].

Mutations, deletions, and aneuploidies that arise during development constitute a second mechanism for developing RPE mosaics. Examples include the pink-eyed dilution gene in the mouse [15,18,19] and the mosaicism observed with a deletion of the human ortholog of the same gene on chromosome 15q [19].

X chromosome inactivation is a third mechanism. This phenomenon arises during development as one member of a complement of two X chromosomes in the female is inactivated to achieve dosage compensation with respect to the male genotype [20]. Because this selection is random and occurs during development, the final product is an RPE monolayer that is a mosaic with respect to the identity of the X chromosome.

Individual populations of epithelial cells that are apparently genetically identical can, however, also exhibit differences in phenotype. This is termed phenotypic variation or phenotypic heterogeneity [2,3,21–24]. Variegation is another term frequently used to denote phenotypic variation related to chromatin structure [25]. The fundamental distinction between mosaicism and phenotypic variation is that mosaicism refers to heterogeneity of genotype, while phenotypic variation refers only to variation of phenotype. Phenotypic variation can occur in both mosaics and also in genetically homogeneous cell populations. Some authors use “mosaic” and “mosaicism” to describe patterns of phenotypic variation in the RPE. Studies from our own laboratory reported phenotypic variation in the expression of heme oxygenase-1, catalase, and insulin-like growth factor binding protein 2 in the human eye [26,27]. Although we and others use “mosaic” to describe these phenotypic patterns, this heterogeneity may or may not be mosaicism based on the genetic definitions given above. Because we observed only the phenotype of these cells with respect to the levels of protein or mRNA, the term phenotypic variation might be more appropriate.

Nongenetic mechanisms that lead to phenotypic variation are less understood. The mechanisms leading to phenotypic variation in a population of cells with an identical genotype involve epigenetic modification of the genome and the noise that is inherent to gene expression [23,24,28–30]. The epigenetic modifications primarily determine changes in chromatin dynamics in individual cells.

Even in the total absence of epigenetic effects, however, populations of cells with identical genomes can still exhibit phenotypic variation due to transcriptional noise [28]. This effect was first shown by measuring levels of fluorescence for two different reporter genes with identical promoters in a genetically homogeneous cell population [23,24,28–32]. Studies of this type of noise have led to interesting hypotheses concerning the roles that noise in gene expression may play in aging [33].

Given the complexity of mosaicism and phenotypic variation in the RPE, it is important to study these phenomena using a method that can quantify this variation. Visual interpretation of phenotypic variation based on immunohistochemistry is subject to observer bias and also has a narrow dynamic range of signal detection. A more useful method should meet several criteria. The method should be capable of analyzing archival tissue; the vast store of human archival samples is clearly orders of magnitude larger than any other source. The method must also be capable of measuring relative concentrations of mRNA or protein over a large dynamic range of expression; these represent the two most common macromolecules (i.e., mRNA, protein) used to quantify gene expression. Quantification on an individual cell basis is also essential; locations of individual cells must be acquired to reconstruct histological context. Finally, the method should be capable of measuring relative levels of several macromolecules in the same sample.

**Figure 3:**
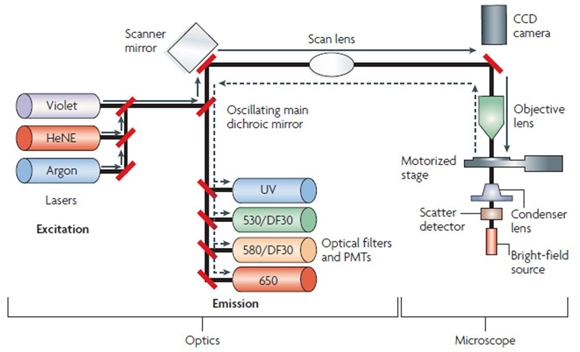
Laser scanning cytometer schematic diagram. The laser scanning cytometer consists of an optics unit that generates the laser scanning beam, an upright epifluorescence microscope with a motorized stage to allow generation of sample scan images, and a computer to acquire and analyze scan data using WinCyte software. Fluorescence is excited by laser sources consisting of an argon laser (blue light; 488 nm), a helium–neon laser (HeNe; red light; 633 nm), and a violet diode laser (405 nm) [35].

Flow cytometry is an extensively used analytical procedure that satisfies most, but not all, of these criteria. The flow cytometric approach is confined to cells in suspension. This process, therefore, is not capable of measuring the position of each cell in its original histological context. LSC is an extension of flow cytometry that performs the same type of analysis on tissue sections and recovers the location of each individual cell. The first literature review on the development and performance of this instrumentation was published in 1997 [34]. A more recent review was published in 2007 [35]. The apparatus consists of one or more lasers used to excite individual fluors, a high precision mechanically driven stage, and detectors that fit the excitation spectra of the fluors being used. The schematic diagram of an LSC in Figure 3 was taken from the literature [35]. The raw data are collected and then processed in a manner similar to computation associated with flow cytometry. We reasoned that LSC should be a useful tool for quantifying phenotypic variation in the RPE. The data presented in this study represent our initial attempts to evaluate LSC for this purpose.

## Methods

### Animals

Mice were purchased from either the National Institute on Aging, Bethesda, MD and Jackson Laboratories, Bar Harbor, ME. Animals were housed under a 12h:12h light–dark cycle. Animals were provided with water and a standard rodent chow (Rodent Diet 5001; PMI Feeds, Inc., Richmond, IN) ad libitum. All procedures conformed to the ARVO Statement for the Use of Animals in Ophthalmic and Vision Research. These procedures were also authorized by the Institutional Animal Care and Use Committee at the University of California, Davis, CA.

### Human tissues

Formalin fixed, paraffin-embedded, archived, donor human eye tissue was graciously provided by Dr. William Lloyd from the Department of Ophthalmology, University of California, Davis. Human donor posterior poles fixed in buffered formalin were obtained from the Foundation Fighting Blindness (Owings Mills, MD). A 9-mm macular punch was dissected from each pole and then embedded in paraffin. Table 1 provides a summary of the mouse and human tissue samples used in this study.

**Table 1:** Mouse and human eye samples used in this study.

**Sample ID**	**Species**	**Gender**	**Age**
070905A	Murine (BALB/c)	Male	24 months
070905D	Murine (BALB/c)	Male	24 months
052705F	Murine (BALB/c)	Male	6 months
052705B	Murine (BALB/c)	Male	6 months
020204A	Murine (C57BL/6)	Male	7 months
E04–019A2	Human	Male	14 months
FFB 766-X	Human	Female	64 years

### Tissue processing for mouse eyes

Each individual mouse was euthanized with gaseous CO_2_, after which the entire blood volume was replaced by perfusion with cold Hanks balanced salt solution containing 1 IU heparin/ml [36]. When the perfusate became clear, the perfusion solution was switched to cold phosphate-buffered 4% paraformaldehyde. After perfusion with 40–50 ml of fixative, individual globes were removed and immersed in the same fixative overnight at 4 °C. The cornea and lens were removed before embedding the posterior pole in paraffin.

### Immunohistochemistry

Paraffin blocks were sectioned on a Leica RM2125RT microtome (Leica, Nussloch, Germany) at 6 µ and then mounted on Fisherbrand SuperFrost Plus glass slides (Fisher Scientific, Houston, TX). After paraffin removal, sections from fixed human globes were processed for antigen retrieval using 1X Dako Target Retrieval solution (Dako, Carpinteria, CA) for 30 min at 98 °C. Slides were rinsed with PBS (Applied Biosystems, Austin, TX, catalog #AM9624), blocked with PBS containing 3% BSA (Jackson ImmunoResearch, West Grove, PA), and incubated with primary antibody diluted in blocking buffer overnight at 4 °C. Negative controls were incubated in normal goat IgG (Santa Cruz Biotechnology, Santa Cruz, CA) at the same concentration as its isotype-matched primary antibody. Slides were washed and incubated with a biotinylated secondary antibody (Vector Laboratories, Burlingame, CA) as per the manufactures instructions, washed, and incubated with the Vectastain ABC Reagent containing the enzyme alkaline phosphatase (Vector Laboratories), and washed once more with PBS before incubating with nitroblue tetrazolium/5-bromo-4-chloro-3-indolyl phosphate (NBT/BCIP; Vector Laboratories). After color development, the slides were washed, counterstained with Nuclear Fast Red (Vector Laboratories), and washed again before dehydration and coverslipping.

### Double immunolabeling

Double immunolabeling of paraffin tissue sections (6 µ) for LSC was performed following a published procedure [37]. Tissue was deparaffinized using xylene, cleared in 100% ethanol, and dried for 5 min in a 50 °C oven. Antigen retrieval was performed using 1X Dako Target Retrieval Solution (Dako) at 98 °C for 30 min, followed by 20 min at room temperature. Slides were rinsed 5 min with 0.2× SSC buffer (Promega Corporation, Madison, WI, catalog #G329A), with one change of buffer. Slides were incubated at 37 °C in a humidified chamber for 2 h with 3 μg/ml goat anti-SOD2 polyclonal antibody (M-20 antibody from Santa Cruz Biotechnology, Santa Cruz, CA) and 6 µg/ml of mouse antibovine RPE65 IgG monoclonal antibody (courtesy of Dr. D. Thompson, W.K. Kellogg Eye Center, University of Michigan, Ann Arbor, MI) diluted in immunofluorescence-labeling buffer (1X PBS, 1% fetal bovine serum, and 0.05% Tween-20). Negative controls were incubated with a mixture of goat IgG (Santa Cruz Biotechnology) and mouse IgG_1_ monoclonal antibody (Dako) at the same concentration as its isotype-matched primary antibody. Slides were washed with PBS containing 0.05% Tween-20. Slides were next incubated at 37 °C in a humidified chamber for 2 h with a mixture of secondary antibodies: 7.5 µg/ml Cy5-conjugated donkey antigoat IgG (Jackson ImmunoResearch, West Grove, PA) and 4 µg/ml of Alexa Fluor 488-conjugated donkey antimouse IgG (Molecular Probes, Eugene, OR) and 100 nM 4',6-diamidino-2-phenylindole (DAPI; Molecular Probes) diluted in immunofluorescence-labeling buffer. Slides were then washed in PBS containing 0.05% Tween-20, coverslipped using a solution containing 50% PBS, 50% glycerol, and 100 nM 4',6-diamidino-2-phenylindole, and sealed with rubber cement before analysis. Table 2 lists the antibodies and the suppliers for all reagents used in our current study.

**Table 2:** Primary and secondary antibodies used in this study.

**Primary antibody**	**Secondary antibody**	**Label**
Goat anti-SOD2 IgG (Santa Cruz Biotechnology, Santa Cruz, CA; Cat #sc-18503)	Biotinylated rabbit anti-goat IgG (Vector Laboratories, Burlingame, CA; Cat #BA-5000)	AP
Goat anti-SOD2 IgG (Santa Cruz Biotechnology, Santa Cruz, CA; Cat #sc-18503)	Alexa Fluor 647 conjugated donkey anit-goat IgG (Molecular Probes, Eugene, OR; Cat #A21447)	Long red
Mouse anti-bovine RPE65 monoclonal antibody (Dr. D. Thompson, University of Michigan, Ann Arbor, MI)	Alexa Fluor 488 conjugated donkey anti-mouse IgG (Molecular Probes, Eugene, OR; Cat #A21202)	Green

### Laser scanning cytometry

LSC was performed using a published method originating from our own LSC facility [37]. Slides were scanned with a 40X objective, using the argon, helium-neon, and violet lasers on an LSC (CompuCyte, Cambridge, MA) equipped with filters for green 530/30 nm band pass filter (530/30BP), long-red 650 nm excitation filter long pass (650 EFLP), and blue fluorescence 463/50 nm short pass dichroic filter (DF50). Settings for voltage, photomultiplier tube (PMT), and threshold settings were identical between negative control and experimental samples.

### Data analysis

Raw data from each scan were collected and stored. Analysis was performed using the WinCyte package supplied with the LSC (CompuCyte Corporation, Westwood, MA).

## Results

### The population frequency diagram

**Figure 4:**
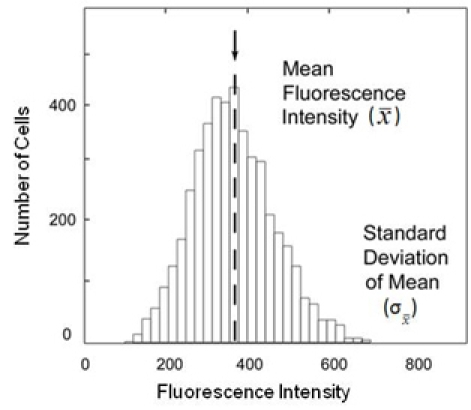
Cytometry population frequency diagram. The *x*-axis represents fluorescence intensity, while the *y*-axis is the number of cells found at each fluorescence level. The labels denote the mean fluorescence intensity ($\bar{x}$) and standard deviation of the mean (σ$\bar{x}$) in the figure [24].

The most fundamental format for displaying data in cytometry is the population frequency diagram. A schematic population frequency diagram is shown in Figure 4. The *x*-axis represents fluorescence intensity, while the *y*-axis is the cell count found at each fluorescence level. For typical experiments, fluorescence is generated by the laser excitation of a fluorescent secondary antibody binding to cells immunolabeled with a primary antibody. Because the data are collected one cell at a time, it is possible to count the number of cells with a given value of immunofluorescence. The *y*-axis, therefore, represents cell number. This figure was adapted from the original article with permission from the publishers [24].

Data for the entire population typically fit a normal distribution. As a result, descriptive statistics can be used to characterize the population. The first of these is the mean fluorescence intensity (MFI) for the entire population of cells; this is denoted as $\bar{x}$ in the figure. If the population does not fit a normal distribution, MFI refers to the median fluorescence intensity; for normal populations the median is identical to the mean. The second is the standard deviation of the mean, denoted as σ$\bar{x}$. The third is the coefficient of variation (CV). The CV is a unitless variable that represents the standard deviation normalized by the mean (CV=σ$\bar{x}$/$\bar{x}$×100). Other measures of variation within the population have been proposed as better descriptors [24], but the definition given here is the most routinely used in cytometry.

The CV is generated by several different factors. The first of these is noise due to section thickness variation, noise in the fluorescence detectors, and uncontrolled variables introduced into the experimental protocol [28]. The sum of all these contributions is termed *extrinsic noise*. The extrinsic noise for a set of experiments is typically assumed to be constant. Noise found in gene expression variation is accounted for by transcription, mRNA processing, mRNA transport, mRNA stability, translation, and degradation of proteins. The aggregate of all these variables is called intrinsic noise. Epigenetic and complex genetic contributions to intrinsic noise have recently been described [23]. The CV is therefore the sum of intrinsic and extrinsic noise for the cells being studied. As a result, the CV is a measure of intrinsic noise in gene expression caused by epigenetics, transcription, RNA processing, translation, and protein turnover.

### Laser scanning cytometry data processing

**Figure 5:**
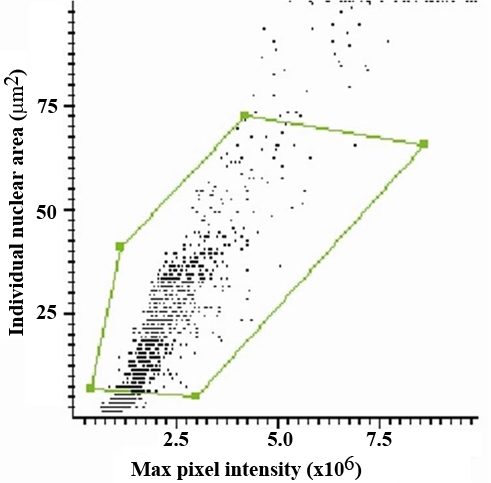
Maximum pixel versus nuclear area scatter plot. A laser scanning cytometry scatter plot of the nuclear area versus the maximum pixel intensity in the blue channel. The polygon defines a gate that separates true nuclei (inside) as opposed to debris and smaller particulates (outside). The gated nuclei are carried forward in the analysis.

LSC is an extension of flow cytometry that performs the same type of analysis on tissue sections and recovers the location of each individual cell. The apparatus uses lasers to excite individual fluorophores, and detectors that fit the emission spectra of the fluors being used. The raw data are collected and then processed in a manner similar to computation associated with flow cytometry. Data are multidimensional and multiparametric and need to be analyzed in a sequential manner. For each experiment the analysis protocol has to be established depending on the question asked and parameters analyzed. At each step, data are displayed looking at two parameters, and the population of cells of interest is identified by a simple process called *gating*. A gate can be represented by a polygon in a scatter plot or a line separating regions with different cell populations in a frequency diagram plot. The common first step in data analysis is to display the data in a scatter plot, where the *x*-axis represents maximum pixel intensity, and the *y*-axis represents the area of the particles detected. A gate is set within the scatter plot to include single nuclei and to exclude doublets and/or debris. In Figure 5 a gate is indicated by the polygon. Only the signals from the gated nuclei within the polygon are considered to belong to “true cells” and are carried forward in the analysis.

### Identification of retinal pigment epithelium cells by immunolabeling with anti-RPE65 antibody

**Figure 6:**
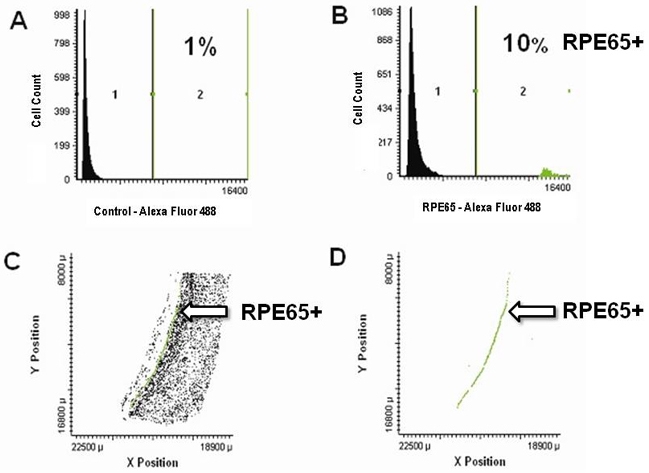
RPE65 immunolabeling of retinal pigment epithelium (RPE) cells. Identification of retinal pigment epithelium cells by immunolabeling with an anti-RPE65 monoclonal antibody using laser scanning cytometry. Panel **A** shows a laser scanning cytometer analysis of a control section (6 μg/ml mouse monoclonal IgG_1_; 4 μg/ml Alexa Fluor 488-conjugated donkey antimouse IgG secondary antibody). Panel **B** shows an anti-RPE65 monoclonal antibody immunolabeled section (6 μg/ml mouse antibovine RPE65 monoclonal primary antibody; 4 μg/ml Alexa Fluor 488-conjugated donkey antimouse IgG secondary antibody). The true RPE65^+^ cells are labeled green. Panel **C** shows an *X–Y* plot of all cells, including RPE65^+^ cells, while panel **D** shows only RPE65^+^ cells (arrows). Abbreviations: anti-RPE65 represents antibody against RPE65 protein, IgG represents Immunoglobulin gamma, RPE65+ represents retinal pigment epithelial cells expressing RPE65 protein.

The unique identification of RPE cells is achieved by immunolabeling with an anti-RPE65 monoclonal antibody. RPE65 is a protein only expressed in the retinal pigment epithelium. Whereas the previous step identified all real cells (Figure 5), this step now identifies which subset of the total cell population is composed of RPE cells. Figure 6 shows a population frequency diagram of a section labeled with a control antibody (Figure 6A) and a population frequency diagram for a section immunolabeled with an anti-RPE65 antibody (Figure 6B). As expected, several weakly fluorescing cells can be found in the population frequency diagram of the section labeled with the control antibody (Figure 6A). To eliminate further analysis of cells with only very weak (background) levels of immunofluorescence, our standard protocol is to set a gate at a fluorescence intensity level that will be greater than the fluorescence intensity levels of at least 95% of the cells exhibiting background labeling (Figure 6A, Region 1). In the analysis of the section immunolabeled with anti-RPE65, cells above this threshold level (Figure 6B, Region 2) are considered to be true RPE65^+^ cells (labeled green). Each cell in the population frequency diagrams in panels A and B is identified with a specific *x* and *y* coordinate within the section that was scanned. An *X–Y* plot of all cells can be constructed (Figure 6C). In this panel, cells from Region 1 (control immunofluorescence) are colored black, and cells from Region 2 (positive immunostaining for RPE65) are colored green. The predominant feature of Figure 6C is a slightly curved line of cells, although several fluorescent cells are scattered within various regions of the *X–Y* plot. When only the cells that are positively immunolabeled for RPE65 (Region 2) are displayed in an *X–Y* plot (Figure 6D), the same image appears. The shape of this feature reflects the position of RPE cells in a section derived from the posterior pole of the mouse eye.

### Double immunolabeling for RPE65 and MnSOD2 in the RPE

It is possible to focus the next steps of the analysis on just the population of RPE cells that are defined by Region 2 in Figure 6B. Immunolabeling for a second antigen found strictly within the population of RPE cells is accomplished with a new primary antibody, a new fluor, and a new laser excitation wavelength. We have chosen MnSOD for this purpose. MnSOD is the product of the SOD2 gene and is located within mitochondria.

**Figure 7:**
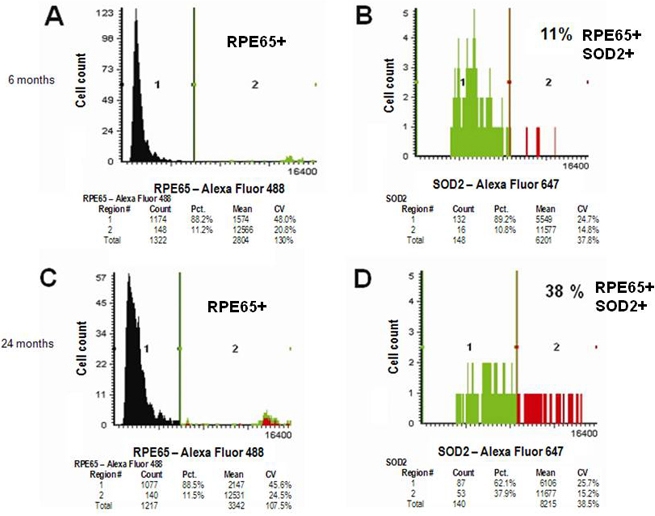
Double immunolabeling for retinal pigment epithelium-specific protein 65 kDa (RPE65) and Mn-superoxide dismutase (MnSOD). Detection of retinal pigment epithelium cells double immunolabeled for Mn-superoxide dismutase and RPE65 in sections from the posterior pole of a 6- and 24-month-old BALB/c mouse by laser scanning cytometry. Panel **A** shows retinal pigment epithelium RPE65^+^ cells for the 6-month-old animal. Panel **B** shows Mn-superoxide dismutase MnSOD^+^/RPE65^+^ double-immunopositive cells for a 6-month-old animal. Panel **C** shows RPE65^+^ cells from a 24-month-old animal. The red color in region 2 represents RPE cells that are RPE65^+^MnSOD^+^, while green represents RPE cells that are RPE65^+^MnSOD^-^. Panel **D** shows MnSOD^+^/RPE65^+^ double-immunopositive cells. In each panel, fluorescence intensities are divided between two regions (1 and 2) defined by a gate at the indicated fluorescence intensity. Each panel shows statistics, including cell counts in each region (Count), total cell count (Total), percentage of cells in each region (Pct.), Mean (MFI), and coefficient of variation (CV). (Primary antibodies: 6 μg/ml mouse monoclonal antibovine RPE65 and 3 μg/ml goat anti-SOD2; Secondary antibodies: 4 μg/ml Alexa Fluor 488-conjugated donkey antimouse IgG and 40 μg/ml Alexa Fluor 647-conjugated donkey antigoat IgG). Abbreviations: RPE65^+^ represents cells expressing RPE65 protein, MnSOD^+^/RPE65^+^ represents cells expressing both Mn-superoxide dismutase and RPE65 protein, BALB/c represents BALB/c strain of mice.

LSC data for RPE65 and MnSOD2 double-immunolabeled RPE cells of the BALB/c mouse at 6 (Figure 7A,B) and 24 months (Panels C and D) are given in Figure 7. Figure 7A shows RPE65 immunolabeling for a section from a 6-month-old animal. Figure 7B shows double immunolabeling for MnSOD for cells within Region 2 in panel A (i.e., RPE cells). Figure 7C shows RPE65 immunolabeling for a section from a 24-month-old animal. Figure 7D shows double immunolabeling for MnSOD for cells within Region 2 in Panel C. The mean fluorescence intensities for both young and old animals (statistics in Figure 7B,D) are very similar. The CV is also nearly identical for the data in Figure 7B,D. There is, however, a difference between the two sets of data. Approximately the same number of RPE cells were counted in both analyses (148 versus 140), but the total number of double-immunolabeled cells for RPE65 and MnSOD is very different (16 versus 53).

### Laser scanning cytometry in pigmented RPE cells

**Figure 8:**
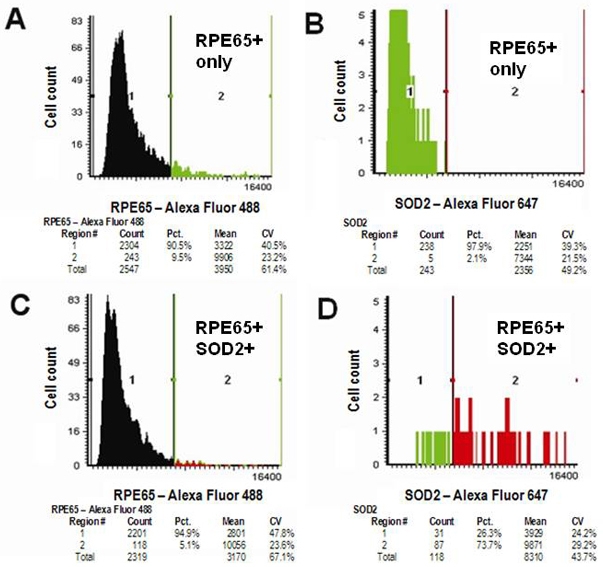
Double immunolabeling in the C57BL/6J mouse. Detection of retinal pigment epithelium cells double immunolabeled for Mn-superoxide dismutase and retinal pigment epithelium-specific protein 65 kDa (RPE65) in sections from the posterior pole of a C57BL/6J mouse by laser scanning cytometry. Panel **A** shows RPE65 immunolabeling in the green channel (6 μg/ml mouse antibovine RPE65 monoclonal antibody; 7 μg/ml Alexa Fluor 488-conjugated donkey antimouse IgG). Panel **B** shows the same section viewed in the long-red channel. Panel **C** shows data from a MnSOD^+^/RPE65^+^ double-immunolabeled section in the green channel. The red color in region 2 represents RPE cells that are RPE65^+^MnSOD^+^, while green represents RPE cells that are RPE65^+^MnSOD^-^. Panel **D** shows the same section in the long-red channel (3 μg/ml goat anti-SOD2 antibody; 40 μg/ml Alexa Fluor 647-conjugated donkey antigoat IgG secondary antibody). Abbreviations: RPE65^+^ represents cells expressing RPE65 protein, MnSOD+ represents cells expressing Mn-superoxide dismutase, MnSOD^+^/RPE65^+^ represents cells expressing both Mn-superoxide dismutase and RPE65 protein, C57BL/6J represents C57BL/6J strain of mice.

All of the previous figures show results from animals with albino eyes. Many mouse strains, such as C57BL/6J, are, however, heavily pigmented. Dense pigment in the RPE and choroid present real issues for light and fluorescence microscopy [38–40]. Figure 8 shows results for quantifying MnSOD in RPE cells using LSC in the C57BL/6J mouse. Panels A and B show population frequency diagrams for sections treated with only the RPE65 antibody (RPE65^+^). In the green channel (Figure 8A) a strong immunopositive population representing all RPE cells can be seen. When the same sample (RPE65^+^) is viewed in the long-red channel (Figure 8B), only background fluorescence is seen. Figure 8C,D show results for RPE65^+^/MnSOD^+^ double immunolabeling. Figure 8C shows cells that are positively labeled for RPE65 in the green channel. Figure 8D shows the double-positive cells (RPE65^+^/ MnSOD^+^). Approximately 74% of the cells identified as RPE cells are also labeled for MnSOD.

The pigment present in the C57BL/6J RPE did cause background interference in the green channel. To minimize background fluorescence with respect to RPE65 immunolabeling, we tested concentrations of 4, 7, and 20 μg/ml Alexa Fluor 488 donkey antimouse IgG-labeled secondary antibody (data not shown). These results indicated that 7 μg/ml of Alexa Fluor 488-labeled secondary antibody is sufficient to separate immunopositive cells from the background fluorescence in the population frequency diagram (data not shown). The same problem was encountered when viewing double-immunolabeled cells in the far-red channel. Ultimately, we used 40 μg/ml of Alexa Fluor 647, an upper limit suggested by the manufacturer in both human and pigmented mouse eyes.

### Laser scanning cytometry in human eyes

**Figure 9:**
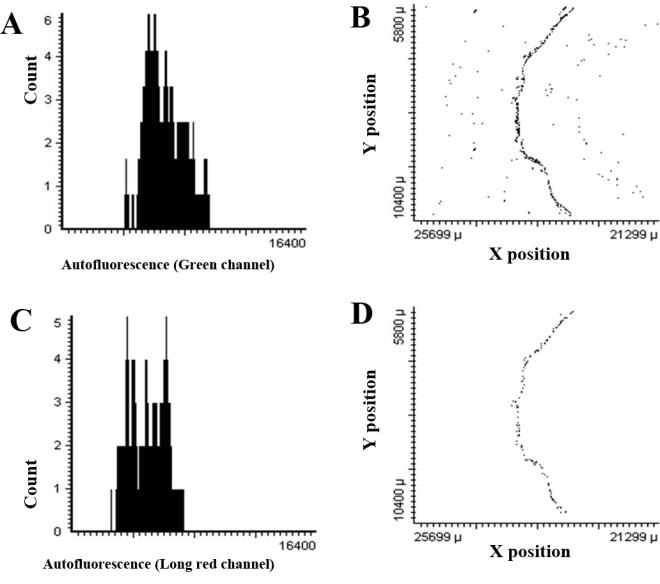
Autofluorescence of human retinal pigment epithelium (RPE). Autofluorescence detection of retinal pigment epithelium cells in the human eye by laser scanning cytometry. A section of the retina from a 14-month-old human male was processed for immunolabeling up to the primary antibody step. The background fluorescence in each channel was then analyzed. Panel **A** shows autofluorescence in the green channel. Panel **B** shows an *X–Y* position plot of the same data. Most autofluorescent cells fall in a line consistent with the location of the retinal pigment epithelium (RPE). Panel **C** shows autofluorescence in the long-red channel. Panel **D** shows an *X–Y* plot of the same data; autofluorescent cells are strictly located within the line of the presumed RPE cells.

The previous data were all acquired in the mouse. Human eyes present the additional complication of autofluorescent materials that are found in the human RPE/Bruch’s membrane. To investigate the extent of autofluorescence in each channel, we performed LSC on sections of a formalin-fixed eye from a 14-month-old male child. Figure 9A shows the population frequency diagram of autofluorescence in the green channel. When these same data are presented in an *X–Y* plot (Figure 9B), the autofluorescence is largely confined to a single line of cells, consistent with reports for autofluorescence in the RPE. There are, however, many autofluorescent cells in the sections that do not appear to be RPE cells. The population frequency diagram of autofluorescence in the long-red channel is shown in Figure 9C. When the data from Figure 9C are viewed in an *X–Y* plot (Figure 9D), the autofluorescence appears to be confined to a single line of cells, consistent with autofluorescence in RPE cells.

### Age-related changes in MnSOD in the human RPE

**Figure 10:**
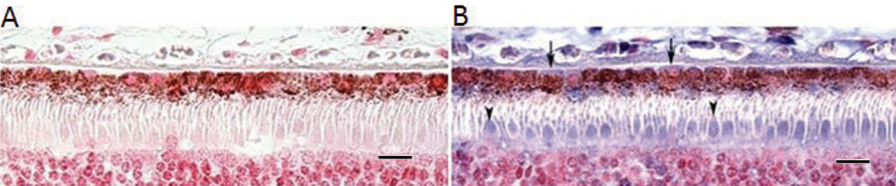
Immunolabeling for Mn-superoxide dismutase (MnSOD) in human retinal pigment epithelium (RPE). Panel **A** shows a negative control section. Panel **B** shows a section from the macula of a 64-year-old female; the section was processed for immunohistochemistry with 4 μg/ml goat antibody against Mn-superoxide dismutase protein (anti-SOD2) primary antibody, 7.5 μg/ml biotinylated rabbit antigoat IgG, avidin/biotin complexed with alkaline phosphatase, and NBT/BCIP substrate. Arrow heads point to cone photoreceptors, and arrows point to RPE cells. The magnification bar represents 10 µ (400×). Abbreviations: NBT represents 4-Nitro blue tetrazolium, BCIP represents 5-bromo-4-chloro-3-indolyl-phosphate.

Figure 10 shows an alkaline phosphatase immunohistochemical labeling for MnSOD in a section of a human posterior pole. Note the variable labeling of RPE cells for MnSOD and the especially bright labeling of cone photoreceptors (Figure 10B).

**Figure 11:**
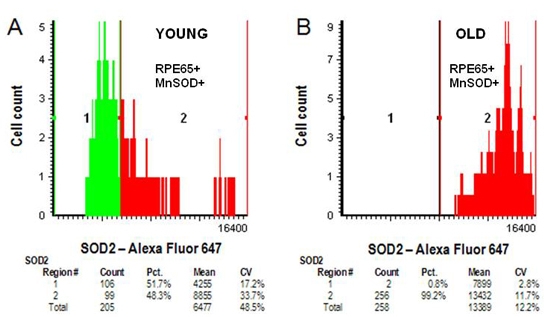
Double immunolabeling for young and old retinal pigment epithelium (RPE). Detection of retinal pigment epithelium cells double immunolabeled for Mn-superoxide dismutase (MnSOD) and retinal pigment epithelium-specific protein 65 kDa (RPE65) proteins in sections of human eyes at 14 months and 64 years by laser scanning cytometry. Formalin-fixed paraffin-embedded sections were prepared and processed for laser scanning cytometry from a 14-month-old male and a 64-year-old female eye. Panel **A** shows data for the 14-month-old eye double immunolabeled for MnSOD and RPE65 (6 μg/ml mouse monoclonal antibovine RPE65, 4 μg/ml goat anti-SOD2 primary antibodies and 4 μg/ml Alexa Fluor 488-conjugated donkey antimouse IgG and 40 μg/ml Alexa Fluor 647-conjugated donkey antigoat IgG secondary antibodies). Panel **B** shows a similar analysis of a section from a 64-year-old female eye.

Figure 11 presents LSC data for RPE65 and MnSOD double immunolabeling of human posterior poles at 14 months (Figure 11A) and 64 years (Figure 11B) of age. Alexa Fluor 488 was used at 4 μg/ml. Because of substantial autofluorescence in the long-red channel, the concentration of Alexa Fluor 647 was elevated to 40 μg/ml. The statistical analyses of the data are included in each panel. For this sample of two eyes, the MFI increased with age, as did the percentage of RPE cells immunolabeled for MnSOD. The CV, however, decreased from a value of 33.7% in the young eye to 11.7% in the older eye.

## Discussion

The goals of our present study were to develop a method of quantifying phenotypic variation at the protein level in RPE cells within their original histological context and to evaluate how well the method works in the mouse and human species. Our results confirm that LSC is a useful approach to develop the methodology and that the effects of possible confounders, such as pigmentation and autofluorescence, can be minimized. These findings, however, do not address the major reason for attempting to develop this method. Our laboratory is currently studying age-related epigenetic regulation of gene expression, and we have explored LSC as a means of quantifying the phenotypic variation of MnSOD levels found in individual RPE cells.

What can be learned about the mechanisms that generate phenotypic variation using the standard variables obtained from cytometry? These questions have been elegantly addressed in a recent publication [23] in which the authors showed us one way to interpret MFI and CV in biologic terms. The approach is generically referred to as the “two reporter fluorescence” method originally developed by Ellowitz et al. [32]. Two transgenes are designed such that both have the same promoter structure but have slightly different, although nearly identical, reporter sequences. When the reporter genes are nearly identical, influences of message stability, differential translation, or protein stability can be assumed to not impact the experimental data. Through several experimental steps of sequential transductions and clonal expansions, the authors produced a set of clonal sublines where each transgene had only one integration site. When flow cytometry is performed on each of these sublines, the fluorescence level of each transgene can be quantified for the total population of cells analyzed.

The following generalizations were made concerning experiments in the study described above. Because the promoters for each transgene have exactly the same structure, changes in the cellular concentration of factors influencing transcription will have the same effect on both transgenes. This is seen as a common change in the CV in the population frequency diagram for the fluorescence output for each reporter. This variation is termed *inherent* or *correlated noise*. When the average level of expression of the transgenes is different, the likely source of the variation is the local chromatin structure. This change of structure is reflected in a change of MFI in the population frequency diagrams of the two transgenes, termed *uncorrelated noise*. As a result of these experiments, it was generally concluded that changes in the state of the chromatin (uncorrelated noise) are reflected by major changes in the MFI of the population frequency diagrams, while changes due to local concentrations of factors within individual cells result in correlated noise as evidenced by synchronous changes in the CV in the population frequency diagrams for both transgenes.

How might these generalizations apply to our observations using LSC? Age-related changes in chromatin structure (such as epigenetic changes of chromatin condensation, histone modifications, or DNA methylation) might mimic position-effect variegation from transgenes. Therefore, when we examine the expression of one gene as a function of a variable, such as chronological age, we might expect age-related changes in the chromatin structure to yield changes in the MFI of a single gene. Because this effect could be highly stochastic with respect to individual cells, the net result could be age-related shifting of the MFI peak as a fraction of the population changes as a function of age. This was, in fact, what we observed for the expression of MnSOD in the human RPE as a function of age and might be termed *age-related variegation*.

On the other hand, when the variation of gene expression is reflected by an increase or decrease in the CV while the mean fluorescence remains constant, the changes are interpreted to be local variation in concentrations of the regulatory factors that bind to the gene promoter. We observed an age-related decrease of the CV for the expression of MnSOD in the human RPE. This would represent a decrease of intrinsic or correlated noise or age-related noise.

The age-related changes in the expression of MnSOD in the mouse RPE were more subtle in terms of the MFI and CV. The MFI was only minimally changed, and the CV remained unchanged. In both the human and mouse, there was a strong age-related increase in the proportion of cells expressing MnSOD.

According to the paper cited above [23], the current understanding of variations in the level of expression for a single transgene focuses on the difference of integration sites within the host cell genome. In some locations, the reporter gene expression is silenced by the local chromatin structure. In other locations the associated chromatin structure can have a variable effect on the level of transcription. This phenomenon is called position-effect variegation [25], where position refers to location within the genome and its associated chromatin structure and variegation refers to phenotypic variation.

The effects of chromatin structure on SOD2 gene expression in human cells have recently been investigated [41]. The chromatin immunoprecipitation and nuclease protection studies from this work point to a mechanism by which changes in the chromatin structure lead to large changes in gene expression. These interpretations follow directly from the measurements of MFI, CV, and the relative distribution of cells in populations expressing different levels of the gene in question.

When the laser scanning cytometer is used instead of routine flow cytometry, the location of cells belonging to different populations is known and can be used in additional analysis. Given two populations of cells, it is possible to quantify the location and size of individual “clumps” of cells [15,42,43]. The geometric mean of the clump size has been used as a variable to describe how “clumpy” a given distribution is. Based on statistical comparisons, a given distribution is random, clumpy, or regular. These classifications may prove useful in discerning whether expression effects are autocrine or paracrine, an important issue in determining the roles that RPE cells may play in photoreceptor degeneration.

The subject of experimental power has not been addressed thus far. Although data from one given animal may be precisely determined with respect to CV, MFI, and the distribution in multiple populations, it will also be important to understand the animal–animal variation in these same factors. It is reasonable to expect variation in these terms across multiple animals, and an adequate determination of sample size will be important to design experiments with adequate experimental power.

In summary, LSC appears to be a robust method for determining variable protein levels in the RPE, which may lead to new experiments to investigate the mechanism of phenotypic variation. The technical improvements in current instruments, including confocal fluorescence microscopy, will help.
